# Few-Shot Personalized Saliency Prediction Based on Adaptive Image Selection Considering Object and Visual Attention†

**DOI:** 10.3390/s20082170

**Published:** 2020-04-11

**Authors:** Yuya Moroto, Keisuke Maeda, Takahiro Ogawa, Miki Haseyama

**Affiliations:** 1 Graduate School of Information Science and Technology, Hokkaido University, N-14, W-9, Kita-ku, Sapporo, Hokkaido 060-0814, Japan; 2 Office of Institutional Research, Hokkaido University, N-8, W-5, Kita-ku, Sapporo, Hokkaido 060-0808, Japan; 3 Faculty of Information Science and Technology, Hokkaido University, N-14, W-9, Kita-ku, Sapporo, Hokkaido 060-0814, Japan; ogawa@lmd.ist.hokudai.ac.jp (T.O.); miki@ist.hokudai.ac.jp (M.H.)

**Keywords:** personalized saliency map, adaptive image selection, multi-task CNN, object detection

## Abstract

A few-shot personalized saliency prediction based on adaptive image selection considering object and visual attention is presented in this paper. Since general methods predicting personalized saliency maps (PSMs) need a large number of training images, the establishment of a theory using a small number of training images is needed. To tackle this problem, although finding persons who have visual attention similar to that of a target person is effective, all persons have to commonly gaze at many images. Thus, it becomes difficult and unrealistic when considering their burden. On the other hand, this paper introduces a novel adaptive image selection (AIS) scheme that focuses on the relationship between human visual attention and objects in images. AIS focuses on both a diversity of objects in images and a variance of PSMs for the objects. Specifically, AIS selects images so that selected images have various kinds of objects to maintain their diversity. Moreover, AIS guarantees the high variance of PSMs for persons since it represents the regions that many persons commonly gaze at or do not gaze at. The proposed method enables selecting similar users from a small number of images by selecting images that have high diversities and variances. This is the technical contribution of this paper. Experimental results show the effectiveness of our personalized saliency prediction including the new image selection scheme.

## 1. Introduction

Many researchers have attempted to predict a saliency map that indicates image components that are more attractable than their neighbors [1,2,3,4]. Since a saliency map reflects human visual attention, it has been expected to contribute to image processing tasks including image re-targeting [5,6], image compression [7,8], and image enhancement [9,10]. The purpose of those studies is prediction of instinctual human visual attention, that is, the common regions of images to humans. Such a saliency map is called a Universal Saliency Map (USM). However, visual attention can differ between persons if individual backgrounds are taken into account [11,12,13]. In fact, since it has been reported that each person views regions of images reflecting personalized interests [14,15,16], the prediction of person-specific visual attention, which is called a Personalized Saliency Map (PSM), has been needed [17,18].

In order to accurately predict PSMs, Xu et al. constructed a PSM dataset and proposed a PSM prediction method [14,19]. Their PSM dataset includes a large number of images and their corresponding gaze data obtained from many persons. To the best of our knowledge, their PSM dataset is the first dataset focusing on PSM prediction. Xu’s method, which is based on a multi-task Convolutional Neural Network (multi-task CNN) [20], needs a large amount of training data for PSM prediction. Thus, for predicting a PSM by this method for a new person not included in the PSM dataset, a large amount of gaze data, which involve a heavy burden, must be obtained for retraining the multi-task CNN. Furthermore, in a real-world situation, PSM prediction of new images not included in the PSM dataset is necessary. Thus, a PSM prediction method without such large-scale data acquisition is desirable. Our previous study revealed that the use of gaze data obtained from similar persons, who view regions in images similar to those viewed by the target person, is effective for PSM prediction [21]. Since the previous study assumes that similar persons have already gazed at the new image, the actual gaze data of similar persons can be utilized. However, since the new image is not always gazed by similar persons, a method that can estimate a PSM of the target person based on predicted PSMs calculated from similar persons is a much more practicable approach. From the above discussions, construction of such a method is a challenging but indispensable task to be addressed.

For predicting a PSM for the new target person, he/she needs to view several images to search for similar persons. Before this procedure, we need to select images from the PSM dataset for calculating person similarities between the target person and those included in the PSM dataset. However, if the selected images are visually similar to each other, the calculated person similarities are not reliable. In order to realize robust PSM prediction with reduction in the number of selected images, an adaptive image selection scheme solving the above problem is necessary. Specifically, we focus on the following two aspects: 1) diversity of images and 2) variance of PSMs. Since the PSM dataset consists of images that have high diversity, we should also select images with maintenance of their diversity. Moreover, the variance of PSMs for persons included in the PSM dataset should be high since the regions in images that many persons commonly gaze at or do not gaze at can be represented by a USM. Thus, by introducing an adaptive image selection scheme focusing on the above two aspects, it is expected that PSM prediction for the new target person is realized with high accuracy.

**Figure 1:**
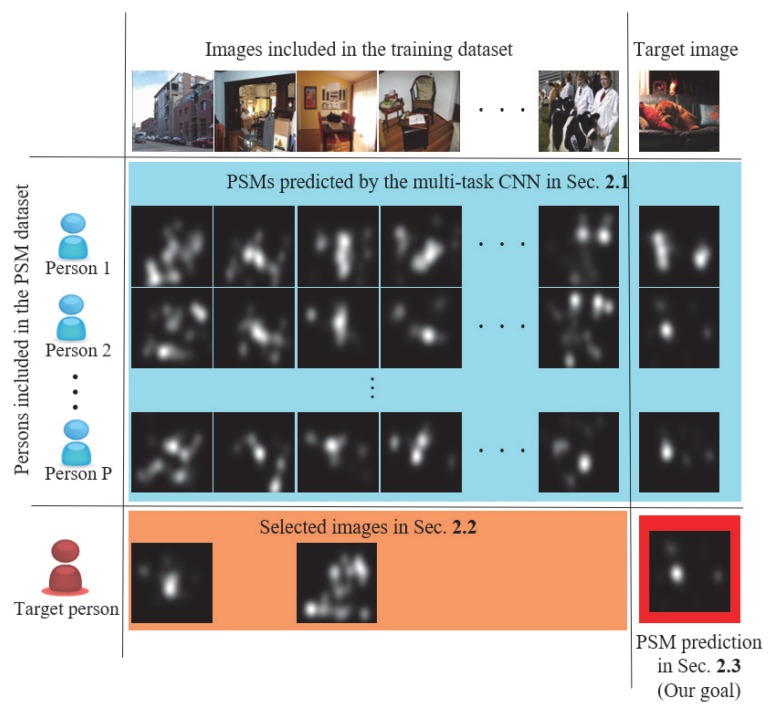
The problem setting of our study. The purpose of our study is Personalized Saliency Map (PSM) prediction of a target person for images not included in the training dataset. For predicting a PSM for a target image, the target person needs to view only some images, which have been viewed by persons included in the training PSM dataset.

This paper presents a few-shot PSM prediction (FPSP) method using a small amount of training data based on adaptive image selection (AIS) considering object and visual attention. Figure 1 shows an illustration of the problem we try to tackle. First, we construct and train a multi-task CNN from the PSM dataset for predicting PSMs of persons included in the PSM dataset [20]. Next, the person similarity is calculated by using selected images included in the PSM dataset. These images are chosen by AIS focusing on the diversity of images and the variance of PSMs. For guaranteeing the high diversity of the selected images, AIS focuses on the kinds of objects included in the training images in the PSM dataset by using a deep learning-based object detection method. Then, objects that have high variances of PSMs are detected, and then we can adaptively select images including such objects, which are shown in the orange area in Figure 1. Finally, FPSP of a target image for the new target person is realized on the basis of the person similarity and PSMs predicted by the multi-task CNN trained for the persons in the PSM dataset. Consequently, FPSP based on AIS for the new target person can be realized from a small amount of training data with high accuracy.

It should be noted that this paper is an extended version of [22]. Specifically, we enable the novel PSM prediction of the target person from those predicted from similar persons based on the multi-task CNN. Furthermore, we newly introduce the AIS into the above PSM prediction approach.

## 2. Few-shot PSM Prediction Based on Adaptive Image Selection

**Figure 2:**
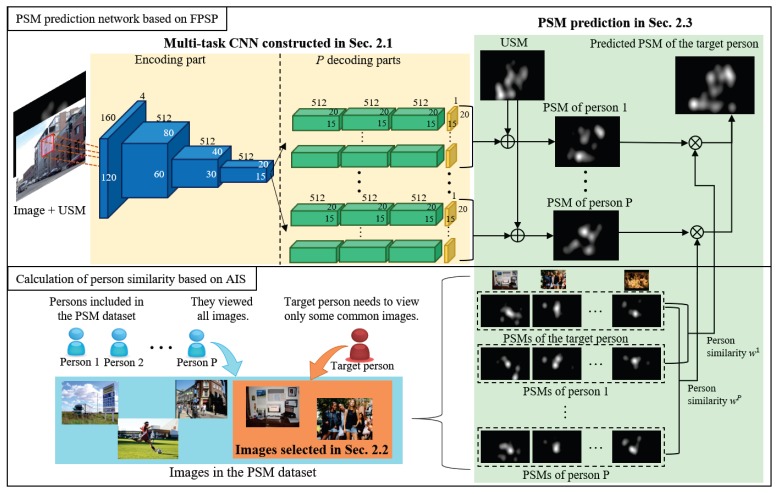
Overview of FPSP and Adaptive Image Selection (AIS). The upper row shows the pipeline of FPSP and the lower row shows the method for the calculation of person similarities based on AIS.

In this section, we explain variables used in this section shown in Table 1 and our proposed method shown in Figure 2. In our method, the multi-task CNN is trained from the PSM dataset for saliency prediction of persons included in the PSM dataset (See Section 2.1). Then, we chose images based on AIS from the PSM dataset (See Section 2.2), and the target person needs to view only the selected images for his/her PSM prediction. Finally, we predict the target person’s saliency map for the new target image by using the predicted saliency maps of similar persons in the PSM dataset (See Section 2.3).

The rest of this paper is organized as follows. In Section 2, FPSP including the AIS scheme is explained in detail. In Section 3, the effectiveness of our proposed method is shown from experimental results. Finally, in Section 4, we conclude this paper.

### 2.1. Construction of a Multi-Task CNN for PSM Prediction

**Figure 3:**
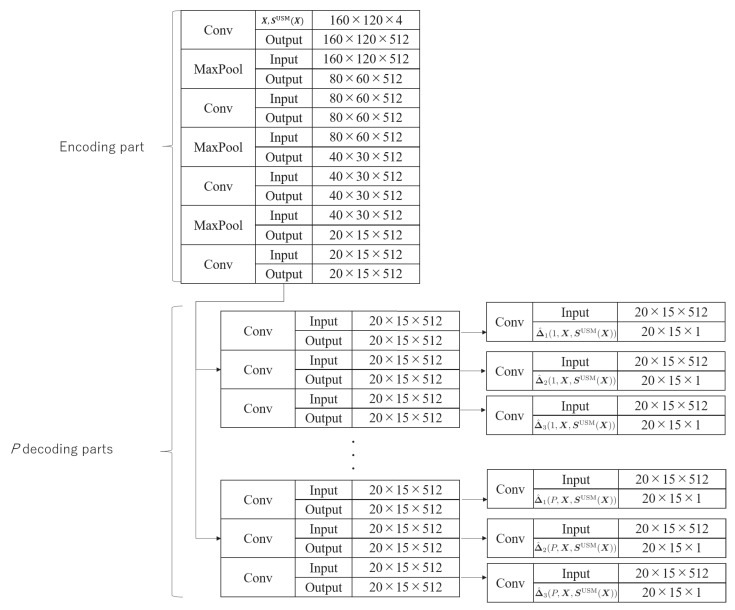
The details of the multi-task CNN used in our method. In this figure, “Conv” and “MaxPool” mean applying a convolution and maxpooling layer to each input data, respectively.

In this subsection, we explain the construction of a multi-task CNN for PSM prediction. This multi-task CNN is constructed for calculating *P* PSMs, where *P* is the number of persons, who are those included in the PSM dataset [20]. In the proposed method, the input data including images $X_{n}\in R^{d_{1}\times d_{2}\times d_{3}}$ (n=1,2,⋯,N; *N* being the number of training images, $d_{1}\times d_{2}$ being the number of pixels, and $d_{3}$ being the number of color channels) and USMs $S^{\mathrm{USM}}\left(X_{n}\right)\in R^{d_{1}\times d_{2}}$ are used for training the multi-task CNN. The USM means the area which many persons gaze at. In our method, the USM $S^{\mathrm{USM}}\left(X_{n}\right)$ can be obtained by an arbitrary method, and it is not our contribution. Thus, it is shown in Section 3. Given PSMs $S^{\mathrm{PSM}}\left(p,X_{n}\right)\in R^{d_{1}\times d_{2}}$ for *P* persons, where $S^{\mathrm{PSM}}\left(p,X_{n}\right)$ is obtained from *p*th person’s gaze data for image $X_{n}$ and included in the PSM dataset [20], we calculate a difference map $\Delta (p,X_{n})$ between the USM and the PSM of each person as $\Delta \left(p,X_{n}\right)=S^{\mathrm{PSM}}\left(p,X_{n}\right)-S^{\mathrm{USM}}\left(X_{n}\right)$ by following [14]. The multi-task CNN has one encoding part and *P* decoding parts consisting of three layers. Its output layer provides *P* results of $\Delta \left(p,X_{n}\right)\;\left(p=1,2,...,P\right)$. The detail of multi-task CNN is shown in Figure 3. Moreover, we train the multi-task CNN by minimizing the following loss function:

(1) $$\begin{array}{c} \sum_{l=1}^{3}\sum_{p=1}^{P}\sum_{n=1}^{N}\left|\right|{\hat{\Delta }}_{l}\left(p,X_{n},S^{\mathrm{USM}}\left(X_{n}\right)\right)-\Delta \left(p,X_{n}\right){\left|\right|}_{F}^{2}, \end{array}$$

where ${\hat{\Delta }}_{l}\left(\cdot \right)$ is a difference map calculation function that applies a 1×1 convolution layer to the outputs obtained from *l*th decoding layer, and $\left|\right|\cdot {\left|\right|}_{F}^{2}$ means the operator of the two-order Frobenius norm. Given a new target image $X^{\mathrm{tgt}}$, by using the above trained network, we predict the PSM of person *p* as follows:

(2) $$\begin{array}{c} S^{\mathrm{out}}\left(p,X^{\mathrm{tgt}}\right)={\hat{\Delta }}_{3}\left(p,X^{\mathrm{tgt}},S^{\mathrm{USM}}\left(X^{\mathrm{tgt}}\right)\right)+S^{\mathrm{USM}}\left(X^{\mathrm{tgt}}\right). \end{array}$$

Therefore, PSMs of multiple persons can be predicted by the single model based on the multi-task CNN.

### 2.2. Adaptive Image Selection for Reduction of Viewed Images

**Figure 4:**
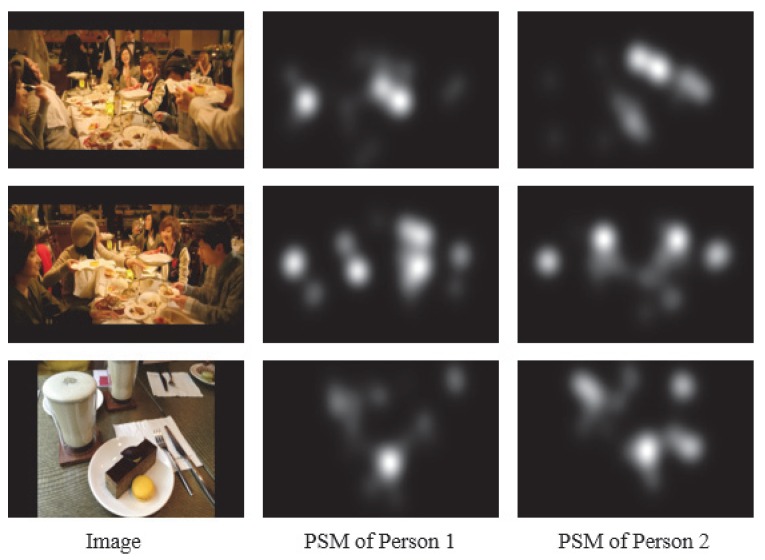
Examples for explaining the diversity of images and the variance of PSMs. Since images in the first and second rows are visually similar, AIS selects either one. On the other hand, for the image in the third row, since PSMs of person 1 and person 2 are similar, AIS does not select this image.

In this subsection, we explain the AIS scheme for the reduction of images that the target person views for predicting his/her PSMs. Given the new person $p^{\mathrm{new}}$ not included in the PSM dataset, the multi-task CNN cannot learn the new person’s PSM since the target person does not gaze at all of the images in the PSM dataset. Therefore, from the target person, we obtain some seed PSMs for images in the PSM dataset [20]. Note that the number of images viewed by the target person should be small for reducing his/her burden. Thus, the influence of one image on training is large, and the diversity of images significantly depends on its selection scheme. Although the PSM dataset has diversity of images, selected images do not necessarily have high diversity. We therefore propose a novel image selection method for maintaining the diversity of images with consideration of the kinds of objects in images and the variance of PSMs as shown in Figure 4. For maximizing the kinds of objects included in the selected images, we apply YOLO-v3 [23], which is one of the novel object detection methods, to the images included in the PSM dataset. Moreover, we use gaze data of persons included in the PSM dataset in order to consider the variance of PSMs. In AIS, we select images based on the detected objects and their PSMs. Specifically, we select objects that have high variances of PSMs since objects that have low variances are expected to be represented by a USM. Finally, we select images that include many kinds of objects having a high variance of PSMs. First, we detect objects $O_{\left(n,m\right)}$ (m=1,⋯,M; *M* being the kinds of objects in all images) in images by using YOLO-v3 [23]. Detected objects are represented by the bounding box in which sizes are $d_{\left(n,m\right)}^{h}\times d_{\left(n,m\right)}^{w}$. Then, we calculate the object variance $v_{\left(n,m\right)}$ as follows:

(3) $$\begin{array}{cc} \; & v_{\left(n,m\right)}=\frac{1}{d_{\left(n,m\right)}^{h}\times d_{\left(n,m\right)}^{w}}\sum_{j=1}^{d_{\left(n,m\right)}^{h}}\sum_{k=1}^{d_{\left(n,m\right)}^{w}}\frac{1}{P}\sum_{p=1}^{P}{\left\{S^{\mathrm{PSM}}{\left(p,O_{\left(n,m\right)}\right)}_{\left(j,k\right)}-{\bar{S}}^{\mathrm{PSM}}{\left(O_{\left(n,m\right)}\right)}_{\left(j,k\right)}\right\}}^{2}, \end{array}$$

(4) $$\begin{array}{cc} \; & {\bar{S}}^{\mathrm{PSM}}{\left(O_{\left(n,m\right)}\right)}_{\left(j,k\right)}=\frac{1}{P}\sum_{p=1}^{P}S^{\mathrm{PSM}}{\left(p,O_{\left(n,m\right)}\right)}_{\left(j,k\right)}, \end{array}$$

where $S^{\mathrm{PSM}}\left(p,O_{\left(n,m\right)}\right)$ is the PSM of person *p* for the object $O_{\left(n,m\right)}$, and (j,k) means the pixel location. Note that we treat $v_{\left(n,m\right)}=0$ if image $X_{n}$ does not include *m*th object, and we adopt the largest $v_{\left(n,m\right)}$ if image $X_{n}$ includes *m*th objects. For selecting images, we calculate the sum of variances, ${\bar{v}}_{n}$, of PSMs for each image as follows:

(5) $$\begin{array}{c} {\bar{v}}_{n}=\sum_{m=1}^{M}v_{\left(n,m\right)}. \end{array}$$

Finally, we select *C* images that have the highest values in Equation (5) from the PSM dataset. It is known that human visual attention depends on objects, and our method that explicitly uses this relationship is a simple but useful for maintaining the diversity and the variance. In particular, AIS adopts YOLO-v3 that has achieved remarkable high performance in the field of recent object recognition and enables for calculating the variance of visual attention pixel-wise. Thus, AIS that focuses on the combination use of the object detection and the visual attention is effective.

### 2.3. FPSP Based on Person Similarity

In this subsection, we explain FPSP using person similarity and the predicted PSM by the multi-task CNN. We predict the PSM of the new person $p^{\mathrm{new}}$ based on the similar persons’ PSMs predicted by the multi-task CNN. First, we predict $S^{\mathrm{out}}\left(p,X_{c}^{\mathrm{sel}}\right)$ by inputting the target image into the multi-task CNN and using Equation (2), where $X_{c}^{\mathrm{sel}}$ (c=1,2,⋯,C) are the *C* images selected in Section 2.2. Next, from the predicted PSMs $S^{\mathrm{out}}\left(p,X_{c}^{\mathrm{sel}}\right)$, we calculate cross correlation as a similarity score $\beta^{p}$ between the target person $p^{\mathrm{new}}$ and person *p* included in the PSM dataset as follows:

(6) $$\begin{array}{c} \beta^{p}=\frac{1}{C}\sum_{c=1}^{C}\mathrm{corr}\left(S^{\mathrm{PSM}}\left(p^{\mathrm{new}},X_{c}^{\mathrm{sel}}\right),S^{\mathrm{out}}\left(p,X_{c}^{\mathrm{sel}}\right)\right), \end{array}$$

where corr (·,·) calculates the cross correlation. Note that $S^{\mathrm{PSM}}\left(p^{\mathrm{new}},X_{c}^{\mathrm{sel}}\right)$ is obtained by using the gaze data for the target person $p^{\mathrm{new}}$. This means that the new person $p^{\mathrm{new}}$ needs to view only the selected *C* images to obtain the gaze data for calculating the PSM $S^{\mathrm{PSM}}\left(p^{\mathrm{new}},X_{c}^{\mathrm{sel}}\right)$. Then, for eliminating the influence from dissimilar persons, we only select similar persons based on the selection coefficient $a^{p}$ as follows:

(7) $$\begin{array}{c} a^{p}=\left\{\begin{array}{cc} 1 & \left(\beta^{p}>\tau \right)\\ 0 & (\mathrm{otherwise}), \end{array}\right. \end{array}$$

where τ is a pre-determined threshold value. Finally, by using the similarity score and the selection coefficient, we calculate the person similarity between the new person $p^{\mathrm{new}}$ and person *p* as follows:

(8) $$\begin{array}{c} w^{p}=\frac{a^{p}\beta^{p}}{\sum_{p^{\prime }}a^{p^{\prime }}\beta^{p^{\prime }}}. \end{array}$$

By using the person similarities $w^{p}$ and similar persons’ PSMs predicted by the multi-task CNN, we can simply predict the PSM $S^{\mathrm{FPSP}}\left(p^{\mathrm{new}},X^{\mathrm{tgt}}\right)$ of the new person $p^{\mathrm{new}}$ for the target image $X^{\mathrm{tgt}}$ as follows:

(9) $$\begin{array}{c} S^{\mathrm{FPSP}}\left(p^{\mathrm{new}},X^{\mathrm{tgt}}\right)=\sum_{p=1}^{P}w^{p}S^{\mathrm{out}}\left(p,X^{\mathrm{tgt}}\right). \end{array}$$

Therefore, by using the person similarity $w^{p}$, the proposed method enables the prediction of the PSM of the new person from a small amount of training gaze data.

## 3. Experiment

In this section, the effectiveness of the FPSP based on AIS is shown from results of experiments. Section 3.1 shows the experimental settings, and Section 3.2 shows the performance evaluation and discussion.

### 3.1. Experimental Settings

In this subsection, we explain our experimental settings. We used the PSM dataset [20] that consisted of 1600 images and their corresponding gaze data for 30 persons who have normal or corrected-to-normal vision. The gaze data were obtained when each person gazed at each image for three seconds under free-viewing conditions. Moreover, we calculated PSMs based on gaze data by following [24]. We randomly chose 500 images as test images and used the remaining 1100 images as training images. Moreover, we selected *C* images from the training images and changed the number of the selected images, *C*, in {10,20,⋯,100}. In this experiment, we randomly chose 10 persons as the new target persons in Section 2 and used the remaining 20 persons as those used for the training. We used the PSM calculated on the basis of gaze data as Ground Truth (GT). Moreover, the multi-task CNN was optimized on the basis of stochastic gradient descent [25], and then we set mini-batch size, learning rate, momentum and the number of iterations as 9, 0.00003, 0.9 and 1000, respectively. We experimentally set the threshold value τ to 0.7, where its determination will be investigated in future work. In the proposed method, $S^{\mathrm{USM}}\left(X_{n}\right)$ can be calculated as an average of the visual attention of training 20 persons.

For confirming the effectiveness of FPSP including the image selection scheme, we performed qualitative evaluation and quantitative evaluation. In the quantitative evaluation, we used the difference between the predicted PSM and its GT based on Pearson’s correlation coefficient (CC), Kullback–Leibler divergence (KLdiv), and histogram intersection (Sim) [26] by following [27]. We also performed two kinds of comparative experiments. In the first comparative experiment, for revealing the effectiveness of our PSM prediction method based on a small amount of gaze data, we compared our method with the following four comparative methods that predict the USM chosen from the MIT saliency benchmark [28]:

- A USM prediction method based on low level visual features (Itti) [1]
- A USM prediction method based on a graph approach (GBVS) [2]
- A USM prediction method based on the separation of foreground and background in images (signature) [3]
- One of the state-of-the-art USM prediction methods based on deep learning (SalGAN) [4] which was trained from the SALICON dataset [29].

Moreover, we compared our method with the following two PSM prediction methods using a small amount of gaze data:

- A PSM prediction method based on visual similarities (Baseline1) [30]
- A PSM prediction method based on visual similarities and spatial information (Baseline2) [22].

It should be noted that the above comparative methods were trained by using the selected images since we assume that the target person views only selected images. In the second comparative experiment, for revealing the effectiveness of our image selection method, we compared our method with the following image selection methods:

- Image selection based on visual features (ISVF)
  Images having with a low similarity to those of visual features to other images were selected. We adopted the outputs of the final convolution layer of pre-trained DenseNet201 [31] as visual features.
- Image selection focusing on variance of PSMs (ISPSM)
  Images having a high variance of PSMs included in the PSM dataset were selected.

### 3.2. Performance Evaluation and Discussion

**Figure 5:**
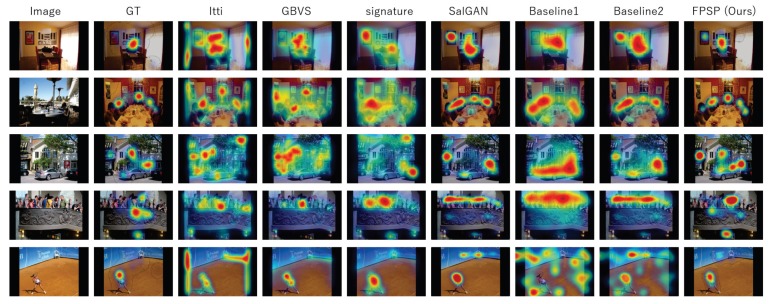
Qualitative results for one person predicted by the FPSP and the comparative methods. In this figure, training images of baselines 1 and 2 and FPSP were selected by AIS.

**Figure 6:**
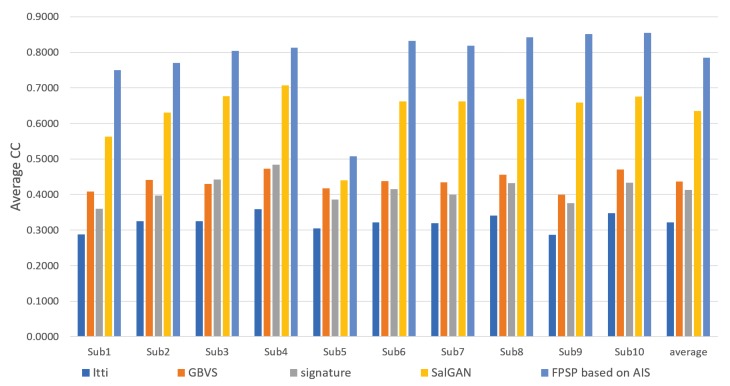
Average CC of each target person (↑) with comparison to USM prediction methods.

**Figure 7:**
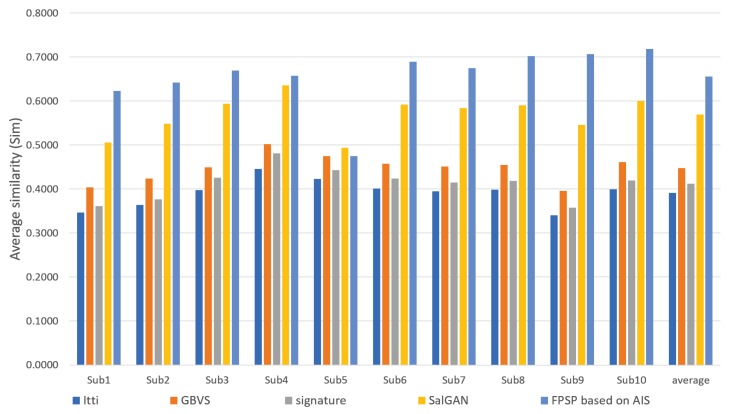
Average similarity (Sim) of each target person (↑) with comparison to USM prediction methods.

**Figure 8:**
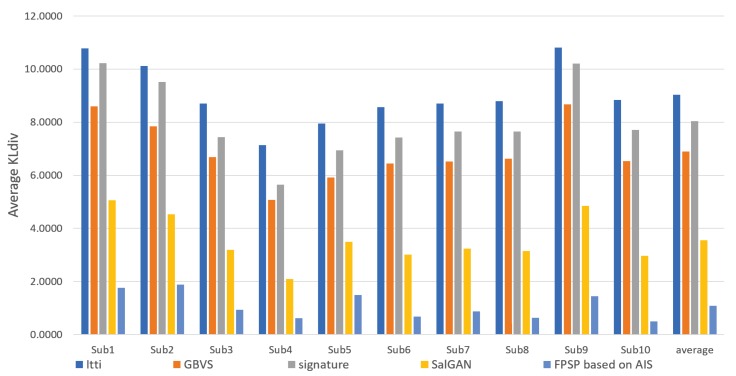
Average KLdiv of each target person (↓) with comparison to USM prediction methods.

**Figure 9:**
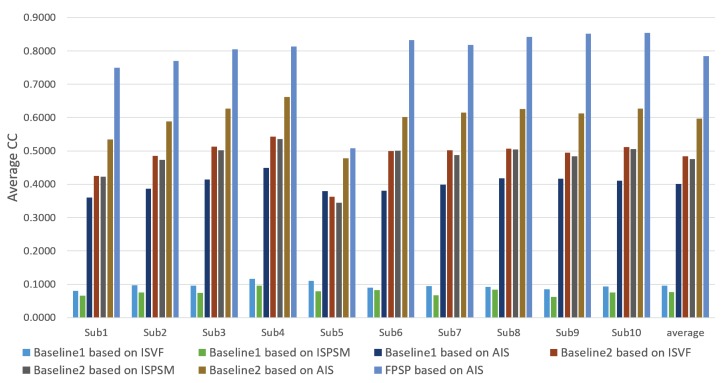
Average CC of each target person (↑) with comparison to PSM prediction methods.

**Figure 10:**
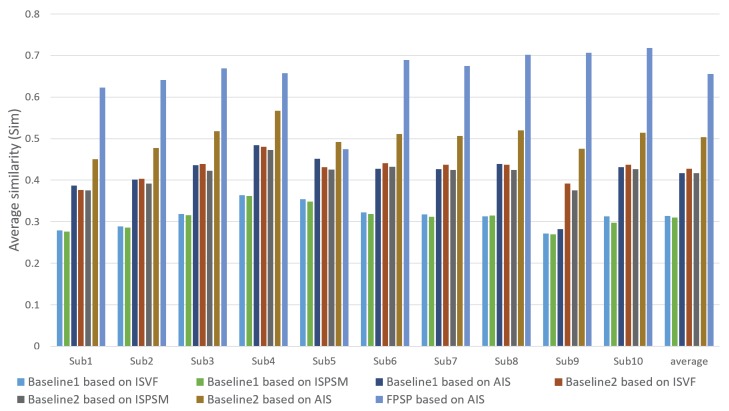
Average similarity (Sim) of each target person (↑) with comparison to PSM prediction methods.

**Figure 11:**
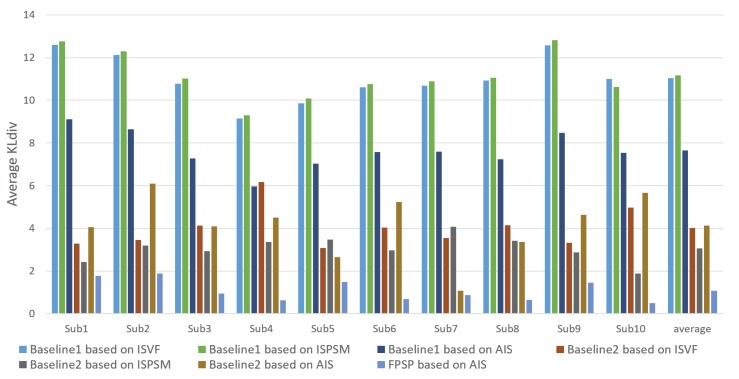
Average KLdiv of each target person (↓) with comparison to PSM prediction methods.

**Figure 12:**
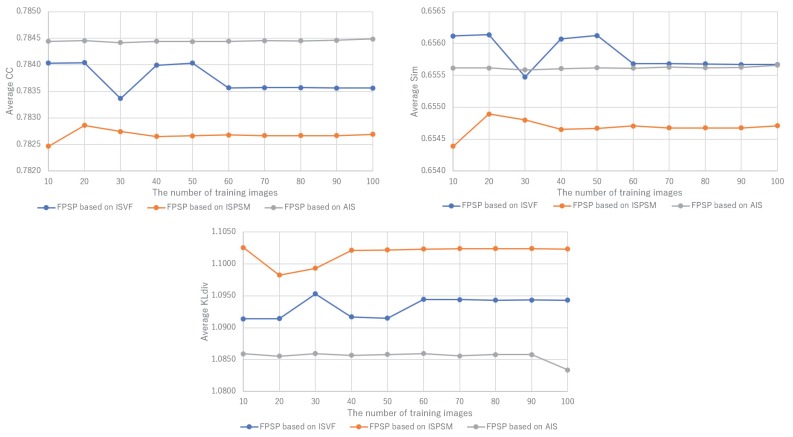
The prediction performance with changes in the number of training images. The robustness of FPSP based on AIS is verified.

In this subsection, we confirm and discuss the experimental results. Figure 5, Figure 6, Figure 7, Figure 8, Figure 9, Figure 10, Figure 11 and Figure 12 and Table 2 shows experimental results. First, Figure 5 shows the predicted results of one person and reveals that the FPSP method enables predicting the PSM that is the most similar to GT among all of the PSMs predicted by comparative methods. In Table 2, we show the average results, and it can be confirmed that FPSP based on AIS is the most effective for the PSM prediction in any evaluation indices. Therefore, by comparing the averages, we confirm the effectiveness of FPSP.

We show the results predicted by FPSP based on AIS and the USM prediction methods for each subject in Figure 6, Figure 7 and Figure 8. Note that we denote 10 target persons as Subs 1–10 in these figures. These figures show that FPSP enables the person-specific prediction for most persons more successfully compared to the USM prediction methods. Specifically, FPSP outperforms SalGAN, which is one of the state-of-the-art USM prediction methods. Thus, we confirm the effectiveness of the construction of the prediction model for each person. Furthermore, we show the results predicted by FPSP based on AIS and the PSM prediction methods for each subject in Figure 9, Figure 10 and Figure 11. These figures show that the results predicted by FPSP are higher than those of other PSM prediction methods. Thus, FPSP enables more accurate prediction than baseline PSM prediction methods. Therefore, the effectiveness of FPSP is verified in the first experiment.

Next, we discuss the difference between AIS, ISVF, and ISPSM in the second experiment. Focusing on the baselines in Table 2, we can confirm that the use of AIS is the most effective image selection method.

Furthermore, Figure 12 shows the performance of FSPS with changes in the number of training images when the training images are selected by AIS, ISVF and ISPSM for the calculation of the person similarity. In CC and KLdiv, the results of FPSP based on AIS are robust to changes in the number of training images and constantly higher than that of AIS and ISPSM. In other words, FPSP based on AIS enables accurately predicting the PSM of the target person just by gazing at 10 images included in the PSM dataset. Thus, it is convinced that our image selection method, AIS, is also effective for FPSP. Therefore, the effectiveness of FPSP based on AIS is verified by the experimental results.

We summarize the discussions. We confirm the effectiveness of the proposed PSM prediction method, FPSP, in Figure 5 and Table 2 from the perspective of the qualitative and quantitative evaluations by focusing the average results. Moreover, by comparing FPSP with USM prediction methods and baseline PSM prediction methods for each person in Figure 6, Figure 7, Figure 8, Figure 9, Figure 10 and Figure 11, it is verified that FPSP enables the accurate prediction for each person. Finally, Figure 12 confirms the robustness and effectiveness of AIS for FPSP. Therefore, we reveal that FPSP based on AIS enables the accurate prediction with a small number of training images and reduces the burden of persons to obtain their gaze data for the PSM prediction.

## 4. Conclusions

In this paper, we have proposed few-shot personalized saliency prediction based on adaptive image selection considering object and visual attention. FPSP enables the accurate PSM prediction with the small number of training images. Moreover, AIS realizes that the number of images that the new person views becomes smaller. Finally, FPSP based on AIS enables the accurate prediction with the small number of training images and reduces the burden of persons to obtain their gaze data for the PSM prediction. Experimental results showed the effectiveness of our proposed method. 

## Figures and Tables

**Table 1:** The list of variables used in Section 2.

Section 2.1	
$X_{n}$	*n*th image in the training data
$X^{\mathrm{tgt}}$	Target image
$S^{\mathrm{USM}}\left(X_{n}\right)$	Universal Saliency Map (USM) of image $X_{n}$
$S^{\mathrm{PSM}}\left(p,X_{n}\right)$	PSM of image $X_{n}$ for person *p*
$S^{\mathrm{out}}\left(p,X^{\mathrm{tgt}}\right)$	PSM predicrted by multi-task Convolutional Neural Network (CNN) for image $X^{\mathrm{tgt}}$ and person *p*
*P*	Number of person
*N*	Number of images
$\Delta (p,X_{n})$	Difference map between USM $S^{\mathrm{USM}}\left(X_{n}\right)$ and PSM $S^{\mathrm{PSM}}\left(p,X_{n}\right)$
${\hat{\Delta }}_{l}\left(p,X_{n},S^{\mathrm{USM}}\left(X_{n}\right)\right)$	Difference map calculated for image $X_{n}$ and person *p*
*n*	Index of images
*p*	Index of persons
*l*	Index of decoding layers
$d_{1}$	Height of image
$d_{2}$	Width of image
$d_{3}$	Number of color channels
Section 2.2	
$O_{\left(n,m\right)}$	*m*th object including *n*th image
$S^{\mathrm{PSM}}\left(p,O_{\left(n,m\right)}\right)$	PSM of object $O_{\left(n,m\right)}$
${\bar{S}}^{\mathrm{PSM}}\left(O_{\left(n,m\right)}\right)$	Average PSM of object $O_{\left(n,m\right)}$
$d_{\left(n,m\right)}^{w}$	Width of *m*th object included in *n*th image
$d_{\left(n,m\right)}^{h}$	Height of *m*th object included in *n*th image
$v_{\left(n,m\right)}$	Variance of *m*th object including *n*th image
${\bar{v}}_{n}$	Average of $v_{\left(n,m\right)}$
*M*	Kinds of objects in all images included in PSM dataset
*C*	Number of selected images
*m*	Index of objects
*j*	Index of width of pixel location
*k*	Index of height of pixel location
*c*	Index of selected images
Section 2.3	
$\beta^{p}$	Similarity score between a target person and person *p*
$p^{\mathrm{new}}$	Target person
τ	Threshold value for person similarity
$a^{p}$	Selection coefficient for person similarity
$w^{p}$	Person similarity between a target person and person *p*
$S^{\mathrm{FPSP}}\left(p^{\mathrm{new}},X^{\mathrm{tgt}}\right)$	PSM predicted by Few-shot Personalized Saliency Prediction (FPSP)

**Table 2:** Comparison of performance in multiple evaluation indices. The mark (↑) means that the higher the index becomes, the higher the performance increases. Similarly, the mark (↓) means that the lower the index becomes, the higher the performance increases. Note that 100 (=*C*) selected images were used for training in baselines 1 and 2 and FPSP. It should be noted that the bold font represents the highest value in its evaluation index.

Methods	CC↑	Sim↑	KLdiv↓
Itti	0.3218	0.3911	9.0397
Gignature	0.4126	0.4122	8.0410
SalGAN	0.6345	0.5689	3.5597
Baseline1 based on ISVF	0.0953	0.3140	11.029
Baseline1 based on ISPSM	0.0762	0.3100	11.161
Baseline1 based on AIS	0.4013	0.4165	7.641
Baseline2 based on ISVF	0.4842	0.4274	4.014
Baseline2 based on ISPSM	0.4761	0.4170	3.057
Baseline2 based on AIS	0.5972	0.5032	4.133
FPSP based on AIS (Ours)	**0.7845**	**0.6557**	**1.083**
